# Engaging young people in the prevention of noncommunicable diseases 

**DOI:** 10.2471/BLT.16.179382

**Published:** 2016-07-01

**Authors:** Rosanna Baker, Ely Taylor, Skander Essafi, Jordan D Jarvis, Christopher Odok

**Affiliations:** a 57 Wood Lane, N6 5UD, London,England.; b Canberra, Australia.; c International Federation of Medical Students Association, Amsterdam, Netherlands.; d Young Professional Chronic Disease Network, Radford, United States of America.

Noncommunicable diseases (NCDs) are one of the biggest public health challenges of the 21st century.^1^ The social and economic impact of NCDs are threatening progress towards sustainable development. NCDs are the leading causes of death,^2^ causing 16 million premature deaths annually.^3^ Four main groups of noncommunicable diseases – cardiovascular diseases, cancers, chronic respiratory diseases and diabetes – account for 82% of all NCD attributable deaths.^3^ By 2025, the global economic cost from these four groups of diseases is predicted to surpass 51 trillion United States dollars.^4^ Despite a common assumption that NCDs are “diseases of affluence” only affecting people in wealthy nations, NCDs disproportionately affect people in low- and middle-income countries. The probability of dying prematurely from NCD in a low- and middle-income country is four times higher than that in high-income countries.^3^

It is important for young people to understand NCDs and their risk factors. Two-thirds of premature deaths in adults are associated with childhood conditions^5^ or behaviours initiated in youth: over 150 million young people smoke;^5^ 81% of adolescents don’t get enough physical activity;^6^ 11.7% of adolescents partake in heavy episodic drinking^7^ and 41 million children under 5 years old are overweight or obese.^8^ Entrenched behaviours and unhealthy environments ensure that NCDs will continue to affect future generations. 

Many young people today have unprecedented access to information and the capacity to act on that information in shaping their own lives, and determining their own health status.^9^ Young people can contribute in several ways to prevent NCDs. By using new media, young voices can provide a novel perspective on NCD prevention and control by sharing targeted messages on key risk factors and interventions. Young people can engage with different communities and share information about NCDs. 

Young people can lead programmes to promote healthy behaviour, such as community exercise classes, or healthy eating programmes. We can contribute to education and awareness initiatives, to inform the public and decision-makers about health problems and solutions. Young medical and allied health students also have a unique opportunity to get involved. Students on clinical rotations can promote effective disease prevention measures such as tobacco cessation. 

Young people can also advocate for policies and practices that aim to improve NCD prevention and care. In 2011, more than 190 countries agreed on global mechanisms to reduce the avoidable NCD burden including a *Global action plan for the prevention and control of NCDs 2013–2020*.^10^ So far this commitment has yielded insufficient resources and political action to reach the target of 25% reduction in premature NCD mortality by 2025. It is our responsibility to hold our governments accountable for their commitments to the 2011 political declaration on NCDs.

We suggest that all institutions working on NCDs evaluate how they gather and act on the perspectives of young people. NCD prevention should be included in the agenda of international youth groups and integrated in all medical, nursing and allied health curriculums. To reduce the impact of NCDs, the next generation needs to be involved in recognizing and changing the conditions that favour these diseases.
